# The Effects of Nonnutritive Sweeteners on the Cariogenic Potential of Oral Microbiome

**DOI:** 10.1155/2021/9967035

**Published:** 2021-06-24

**Authors:** Jianhui Zhu, Jiaxin Liu, Zhengyi Li, Ranhui Xi, Yuqing Li, Xian Peng, Xin Xu, Xin Zheng, Xuedong Zhou

**Affiliations:** ^1^State Key Laboratory of Oral Diseases & National Clinical Research Center for Oral Diseases, West China Hospital of Stomatology, Sichuan University, Chengdu 610041, China; ^2^Department of Cariology and Endodontics, West China Hospital of Stomatology, Sichuan University, Chengdu 610041, China

## Abstract

Nonnutritive sweeteners (NNSs) are sugar substitutes widely used to reduce the negative health effects of excessive sugar consumption. Dental caries, one of the most prevalent chronic diseases globally, results from a pathogenic biofilm with microecological imbalance and frequent exposure to sugars. Some research has shown that certain NNSs possess less cariogenic potential than sucrose, indicating their putative effect on oral microbiome. To uncover the alterations of acidogenic pathogens and alkali-generating commensals, as well as the biofilm cariogenic potential under the influence of NNSs, we selected four common NNSs (acesulfame-K, aspartame, saccharin, and sucralose) and established single-, dual-, and multispecies *in vitro* culture model to assess their effects on *Streptococcus mutans* (*S. mutans*) and/or *Streptococcus sanguinis* (*S. sanguinis*) compared to sucrose with the same sweetness. The results showed that NNSs significantly suppressed the planktonic growth, acid production, and biofilm formation of *S. mutans* or *S. sanguinis* compared with sucrose in single-species cultures. Additionally, decreased *S. mutans*/*S. sanguinis* ratio, less EPS generation, and higher pH value were observed in dual-species and saliva-derived multispecies biofilms with supplementary NNSs. Collectively, this study demonstrates that NNSs inhibit the cariogenic potential of biofilms by maintaining microbial equilibrium, thus having a promising prospect as anticaries agents.

## 1. Introduction

Sugar is the generic name for sweet-tasting, soluble carbohydrates. For most people, “sugar” refers to the granulated household flavoring to enhance sweetness, whose main ingredient is sucrose, one of the most commonly consumed sugars in human diet [1]. Although sugar maintains an important position in food industry, its excessive consumption has been implicated with several health conditions including obesity, diabetes, cardiovascular diseases, and dental caries [2]. Due to public health efforts to lower sugar intake, the consumption of alternative sweeteners has been increasing globally [3].

Dental caries is one of the most prevalent chronic diseases worldwide, characterized by local destruction of dental hard tissues. It is a multifactorial disease that occurs in the presence of a pathogenic biofilm and frequent exposure to sugars [4]. Considered as a highly cariogenic substrate, sucrose can be utilized by pathogenic bacteria to generate acid by-product which ultimately leads to demineralization, as well as extracellular polysaccharides (EPS) which facilitate its colonization and form a protective environment against host defense [5]. From an ecological perspective, the interactions between acidogenic/aciduric bacteria (e.g., *Streptococcus mutans* and *Lactobacillus* spp.) and alkali-generating commensals (e.g., *Streptococcus sanguinis* and *Streptococcus gordonii*) in dental plaque are closely associated with the initiation and development of caries [6, 7].

Sugar substitutes have been identified to contribute in caries prevention, as many of them cannot be utilized efficiently by oral bacteria to produce acids [8]. Certain sugar substitutes have been reported to possess less cariogenic potential than sucrose in *in vitro* biofilm models established on glass beads or enamel slabs, with one or more bacteria strains inoculated [9–11]. However, their effects on the antagonism between acidogenic and alkali-generating bacteria based on saliva-derived multispecies biofilm model have not yet been investigated. Alternative sweeteners can be categorized into nutritive sweeteners (also called bulk or caloric sweeteners) and nonnutritive sweeteners (also called intense or noncaloric sweeteners) according to their different ability to provide energy. Nonnutritive sweeteners (NNSs) are many times sweeter than equally weighted sugar while contributing few or no calories, and are commonly used in a wide range of foods [12].

This study is aimed to assess the effect of four NNSs (acesulfame-K, aspartame, saccharin, and sucralose) on the cariogenic potential of biofilms, which are extensively applied and considered safe for consumption by the United States Food and Drug Administration (FDA) [13]. We measured the growth, biofilm formation, and acid/EPS production of *S. mutans* and *S. sanguinis* after they were cultured alone or in dual- and multispecies biofilm models and comparatively evaluated the results of NNSs groups with reference to sucrose of the same sweetness.

## 2. Materials and Methods

### 2.1. Materials and Reagents

Brain Heart Infusion (BHI) was purchased from BD Difco (USA). Acesulfame-K, crystal violet (CV), Tris hydrochloride (Tris-HCl), Ethylenediaminetetraacetic acid (EDTA), and lysozyme were purchased from Sigma (USA). Saccharin, sucralose, sodium dodecyl sulfate (SDS), and formamide were purchased from J&K scientific (China). Aspartame was purchased from APExBIO (USA). Alexa Fluor 647-labeled dextran conjugate, SYTO 9 green fluorescent nucleic acid stain (485/498 nm), and phosphate-buffered saline (PBS, 50 mM, pH 6.8) were purchased from Thermo Fisher Scientific (USA).

### 2.2. Bacterial Culture

*S. mutans* UA159 and *S. sanguinis* ATCC10556 were commercially obtained from the American Type Culture Collection (ATCC). *S. mutans* and *S. sanguinis* were grown at 37°C and 5% CO_2_ in BHI medium. They were cultured overnight and adjusted to optical density (OD) 0.5-0.6 at 600 nm (1 × 10^8^ CFU/ml based on the OD_600nm_ versus CFU/ml curve of each strain) and further 1 : 100 diluted in the growth medium for inoculation.

### 2.3. Preparation of Different Growth Media

The liquid medium containing one of the NNSs (acesulfame-K, saccharin, aspartame, or sucralose) was prepared to a concentration which has the same sweetness degree as sucrose according to information published by FDA (Table 1). For planktonic growth or single-species biofilm, BHI without any added sweeteners was considered as the negative control, and BHI with 1% sucrose was considered as the positive control. In the study of dual- and multispecies biofilm, the prepared media for all groups were supplemented with 0.2% sucrose to ensure sufficient biofilm adhesion for further analyses. For instance, the medium of acesulfame-K group contained 0.2% sucrose and acesulfame-K maintaining sweetness comparable with 0.8% sucrose. BHI with 0.2% sucrose was considered as the negative control, and BHI with 1% sucrose was considered as the positive control. All of the media were filter-sterilized and stored at 4°C before use.

### 2.4. Planktonic Growth of *S. mutans* and *S. sanguinis*

Overnight cultures of *S. mutans* and *S. sanguinis* were inoculated separately in six sterile 15 ml centrifuge tubes, each of them containing 5 ml different growth media (BHI, BHI with 1% sucrose, and BHI with four sweeteners). After incubation at 37°C and 5% CO_2_ for 24 h, we suspended the cultures and measured their OD_600nm_ using Nanodrop One (Thermo Fisher Scientific Waltham, MA, USA) with cuvettes (light path = 10 mm). The cultures were then recollected, centrifuged at 5000 g for 10 min to remove the precipitation. The pH values of culture supernatants were then recorded by pH electrode InLab Expert Pro (Mettler Toledo, Melbourne, Australia).

### 2.5. Crystal Violet Staining of Single-Species Biofilm

The biofilm formation of *S. mutans* or *S. sanguinis* grown in media containing different NNSs was observed after CV staining. Overnight cultures of each strain were inoculated in a 96-well microtiter plate, each well containing 200 *μ*l medium (BHI, BHI with 1% sucrose, and BHI with four sweeteners). After cultivation for 24 h, we aspirated the culture supernatant, washed each well three times with PBS, fixed the biofilms with methanol for 15 min, and stained with 0.1% (*w*/*v*) CV for 10 min. The redundant CV was removed by PBS rinses until the rinse solution appeared colorless. The images of the wells were captured by a stereo microscope (Olympus SZX10, Japan). The biofilms were then destained by adding 200 *μ*l 33% acetic acid to each well and shaking the plate at 37°C (100 r/min) for 30 min. The destaining solution of each well was added to another blank 96-well microtiter plate before the OD_595nm_ value was measured. The experiment was carried out with triplicate samples and replicated three times, with data normalized by the blank wells.

### 2.6. Dual- and Multispecies Biofilm Model

Dual-species biofilm model was established as described previously [14]. Whole unstimulated saliva was collected on ice from 5 healthy adult volunteers who were asked not to eat or drink 2 h before collection. The saliva was pooled and clarified by low-speed certification (2600 g for 10 min). The supernatant was filtered for sterilization and stored at -80°C before use. A 24-well plate with a glass coverslip in each well was used for biofilm culture. We added 500 *μ*l sterile saliva to each well and incubated the plate at 37°C for 2 h to coat the glass coverslips. After the saliva was aspirated, the growth medium was added into each well. (BHI with 0.2% sucrose, BHI with 1% sucrose, and BHI with four sweeteners). Overnight cultures of *S. mutans* and *S. sanguinis* were inoculated on saliva-coated glass coverslips (inoculum ratio = 1 : 1).

Saliva-derived multispecies biofilm model was established according to Guo et al. [15] with modifications. Whole unstimulated saliva was collected on ice from 5 healthy adult volunteers without antibiotic treatment in the last 3 months or any diseases that might influence the saliva composition. Each sample was centrifuged at 2600 g for 10 min and pooled together. To collect a stock of the saliva-derived multispecies bacteria, 1 ml pooled saliva was added into 5 ml BHI and incubated under anaerobic conditions at 37°C for 24 h. The cultures were centrifuged at 14000 g for 3 min, resuspended in fresh BHI with 25% glycerol, and kept at −80°C. Overnight cultures of *S. mutans* and the saliva-derived multispecies bacteria were resuspended into BHI to OD_600nm_ of 0.5-0.6, respectively, and mixed in a 1 : 10 volume ratio. The mixtures were then 1 : 100 diluted in fresh BHI and added to a 24-well plate with sterile saliva-coated glass coverslips.

Both dual- and multispecies biofilm samples were grown at 37°C for 24 h before further analyses. The culture medium was collected to measure pH values after 24 h cultivation.

### 2.7. Bacteria/Extracellular Polysaccharides Staining

The bacteria and their EPS production were determined by bacteria/EPS staining as described previously [16]. Briefly, 1 *μ*M Alexa Fluor 647-labeled dextran conjugate was added to the culture medium at the beginning of incubation. The labeled dextran serves as a primer for Glucosyltransferases and can be simultaneously incorporated during EPS synthesis in biofilm development [17]. The bacterial cells were labeled with 2 *μ*M SYTO 9 green fluorescent nucleic acid stain (485/498 nm) through 15 min staining after cultivation. The imaging was performed using a confocal laser scanning microscope (Olympus FV3000, Japan) equipped with a 60x (1.42 numerical aperture) oil immersion objective lens. The scan speed, photomultiplier detector gain, and pinhole aperture were kept constant for all image stacks. Representative images were demonstrated using Imaris software (v7.0). The quantities of bacteria and EPS were based on 5 image stacks in each group obtained from three independent tests and measured with COMSTAT2 image analysis program [18] as a plugin of Image J software (v1.8) by calculating biomass.

### 2.8. Fluorescence *In Situ* Hybridization

The sequences of oligonucleotide probes in fluorescence *in situ* hybridization (FISH) were either designed using ARB software packages [19] or acquired from previous studies [16]. Universal bacteria probe (5′-GCTGCCTCCCGTAGGAGT-3′), *S. mutans* specific probe (5′-ACTCCAGACTTTCCTGAC-3′), and *S. sanguinis* specific probe (5′-GCATACTATGGTTAAGCCACAGCC-3′) were labeled with Alexa Fluor 405, Alexa Fluor 488, or Alexa Fluor 594, respectively. The biofilms on glass coverslips were fixed in 4% paraformaldehyde for 6 h, rinsed with PBS, and dried. For probe penetration, the biofilm samples were treated with 500 *μ*l lysis buffer (100 mM Tris-HCl, 50 mM EDTA, 30 mg/ml lysozyme, pH 8.0) at 37°C for 20 min. The biofilms were then rinsed, serially dehydrated in ethanol (50%, 80%, and 100%; 3 min each), and dried. For probe hybridization, each bead was covered with 50 *μ*l of hybridization buffer (0.9 M NaCl, 20 mM Tris-HCl, 0.01% SDS, 20% formamide) containing 3 oligonucleotide probes (2 *μ*M each) and incubated at 46°C for 90 min in a closed cassette with a piece of paper towel soaked by hybridization buffer to maintain humidity. Subsequently, the beads were washed with buffer (20 mM Tris/HCl, 5 mM EDTA, 0.01% SDS, 215 mM NaCl), incubated in the washing buffer at 46°C for 15 min, and rinsed in 4°C nuclease-free water. The images were captured and analyzed with similar tools in the EPS staining experiment.

### 2.9. Statistics

The statistical analysis and graphing of the data were performed using the GraphPad Prism software (v8.0), with all data demonstrated as mean ± standard deviations (SD). Group mean values were compared by one-way ANOVA and Tukey post hoc test. A two-tailed *P* < 0.05 was considered to be statistically significant.

## 3. Results

### 3.1. NNSs Suppress the Growth and Acid Production of *S. mutans* or *S. sanguinis* in Planktonic Cultures and Single-Species Biofilm

**Figure 1:**
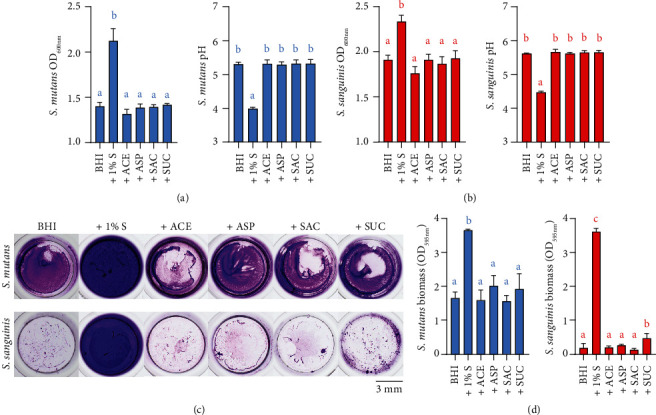
Effects of NNSs on the planktonic growth, acid production, and biofilm formation of *S. mutans* or *S. sanguinis*. OD_600nm_ and pH value measurement of *S. mutans* (a) and *S. sanguinis* (b) planktonic cultures, respectively. (c) Representative images of *S. mutans* and *S. sanguinis* biofilms after crystal violet staining. (d) The quantitative data of single-species biofilm biomass after destaining. Results are presented as mean ± standard deviation. Different lowercase letters indicate a significant intergroup difference and are marked according to mean value. (S: sucrose; ACE: acesulfame-K; ASP: aspartame; SAC: saccharin; SUC: sucralose.).

After 24 h incubation, suspended *S. mutans* cultures containing NNSs exhibited a significantly lower level of OD_600nm_ and a significantly higher level of pH value than that containing 1% sucrose (Figure 1(a)). Similar significant relatively inhibitory effects were observed against *S. sanguinis* planktonic growth and acid production, but to a lesser extent (Figure 1(b)). Notably, these effects in planktonic bacterial cultures displayed no significant difference between each NNS group. Moreover, CV staining demonstrated the decreased biofilm formation of *S. mutans* or *S. sanguinis* cultured with NNSs compared to that with sucrose and the decline of *S. sanguinis* biofilms was more obvious than that of *S. mutans* (Figures 1(c) and 1(d)). The biofilm formed in the sucralose group was significantly increased compared with the negative control while far less than the sucrose group (Figure 1(d)).

### 3.2. NNSs Diminish *S. mutans*/*S. sanguinis* Ratio and Acid/EPS Generation in Dual-Species Biofilms

**Figure 2:**
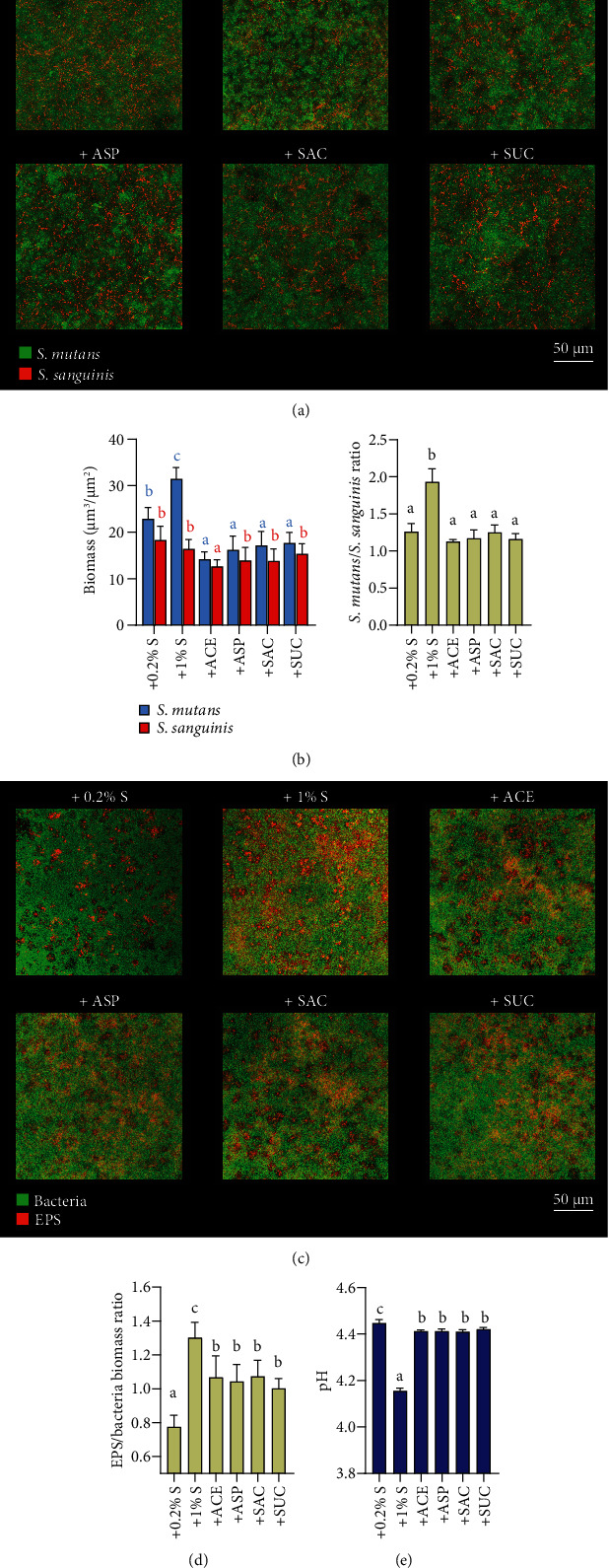
Effects of NNSs on bacterial composition, extracellular polysaccharides (EPS) generation, and acid production of dual-species biofilms. (a) Representative images of dual-species biofilms after fluorescence in situ hybridization. Green, *S. mutans*; Red, *S. sanguinis*. (b) Quantitative data of *S. mutans/S. sanguinis* biomass and bacterial composition. (c) Representative images of *S. mutans/S. sanguinis* dual-species biofilms after EPS staining. Green, bacteria; red, EPS. (d) The quantitative data of EPS/bacterial amount in dual-species biofilms. (e) The pH values of culture media of dual-species biofilms. Results are presented as mean ± standard deviation. Different lowercase letters indicate a significant intergroup difference and are marked according to mean value. (S: sucrose; ACE: acesulfame-K; ASP: aspartame; SAC: saccharin; SUC: sucralose.).

The influence of NNSs on *S. mutans* and *S. sanguinis* was further investigated in dual-species biofilms using FISH and EPS staining. The images of biofilms labeled with species-specific FISH probes showed that NNSs significantly suppressed the absolute biomass of *S. mutans* and the *S. mutans*/*S. sanguinis* ratio compared with the 0.2% sucrose treated control (Figures 2(a) and 2(b)). However, among four NNSs, only the acesulfame-K group exhibited a significant decrease of absolute *S. sanguinis* biomass, although to a lesser degree than *S. mutans*. It is indicated that NNSs selectively constrained the growth of *S. mutans* while keeping the growth of *S. sanguinis*, thus promoting the relative dominance of *S. sanguinis* in dual-species biofilms. Moreover, significant inhibitory effects on EPS production and higher pH value of the culture media were observed in dual-species biofilms with NNSs (Figures 2(c)–2(e)).

### 3.3. NNSs Reduce Acid/EPS Production of Saliva-Derived Multispecies Biofilm and Acesulfame-K Decrease *S. mutans*/*S. sanguinis* ratio

**Figure 3:**
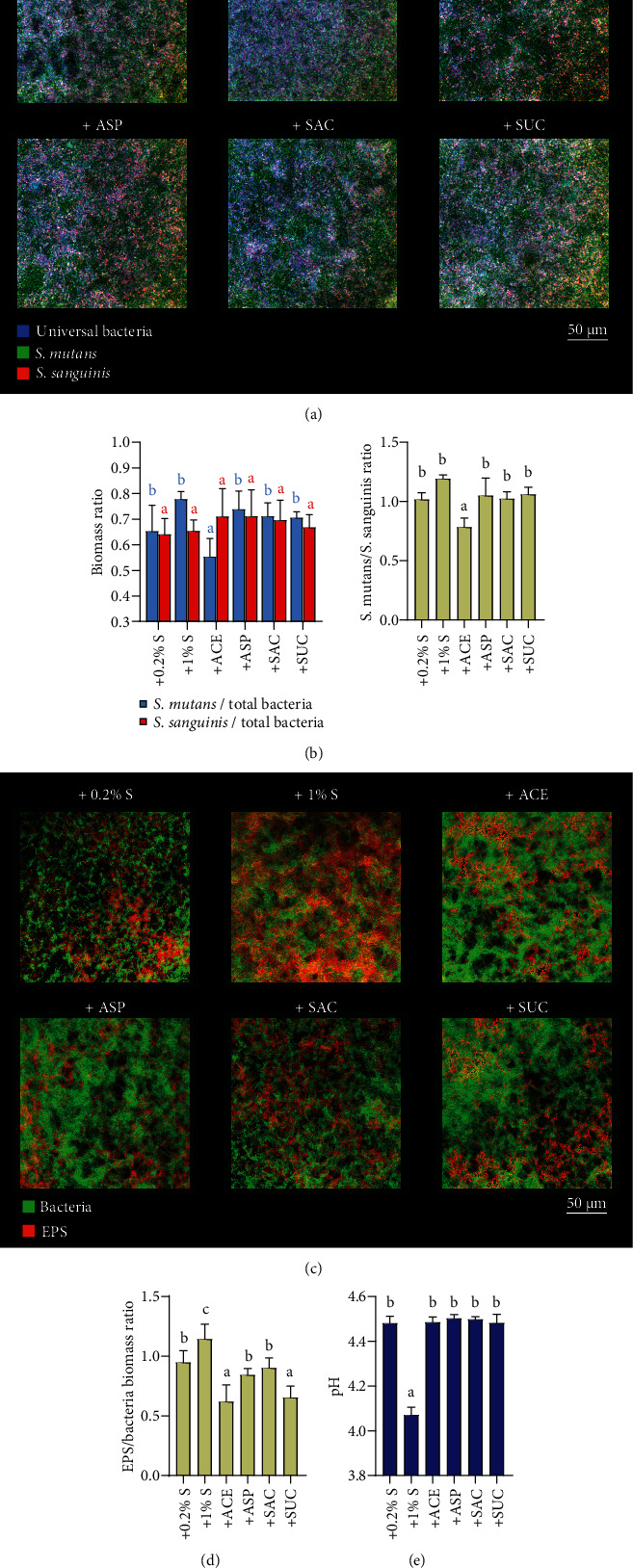
Effects of NNSs on bacterial composition and extracellular polysaccharides (EPS)/acid production of saliva-derived multispecies biofilms. (a) Representative images of multispecies biofilms after fluorescence *in situ* hybridization. Green: *S. mutans*; red: *S. sanguinis*; blue: universal bacteria. (b) Biomass ratio quantitative data of *S. mutans*/universal bacteria (blue), *S. sanguinis*/universal biomass (red) and *S. mutans*/*S. sanguinis* (yellow). (c) Representative images of multispecies biofilms after EPS staining. Green: bacteria; red: EPS. (d) The quantitative data of EPS/bacterial amount in multispecies biofilms. (e) The pH values of culture media of multispecies biofilms. Results are presented as mean ± standard deviation. Different lowercase letters indicate a significant intergroup difference and are marked according to mean value. (S: sucrose; ACE: acesulfame-K; ASP: aspartame; SAC: saccharin; SUC: sucralose.).

We further examined the effect of NNSs on the microbial composition and EPS/acid production of the multispecies biofilms which were derived from saliva and supplemented with *S. mutans* inoculation. Notably, the *S. mutans*/universal bacteria ratio and *S. mutans/S. sanguinis* ratio were significantly lower in biofilms with acesulfame-K than other treated groups (even the 0.2% sucrose group). However, analysis of FISH-labeled multispecies biofilms showed no significant difference among all groups in the biomass ratio of *S. sanguinis* to universal bacteria (Figures 3(a) and 3(b)). These results suggested the particular inhibitory effect of acesulfame-K on typical acidogenic bacteria in a multispecies biofilm. In addition, biofilms developed in media with NNSs exhibited less EPS and acid production compared with 1% sucrose (Figures 3(c)–3(e)).

## 4. Discussion

Dental caries results from polymicrobial infection in which imbalance between acidogenic pathogens and alkali-generating commensals in plaque biofilm plays a crucial role [6, 20, 21]. The acid accumulation and pH decline of microbial biofilm led by its frequent exposure to carbohydrates selectively enrich the acidogenic/aciduric species (e.g., *S. mutans* and lactobacilli) and suppress the less aciduric commensal residents (e.g., *S. sanguinis*), driving the shift of microbial community to a more cariogenic consortium consequently. This positive feedback leads to a continuous local pH decline below the critical value which results in the demineralization of tooth tissues and eventually the development of caries lesion [6, 22]. Sugar substitutes are generally considered to have negative effects on caries initiation [1, 23]. Some research has reported certain sweeteners prominently inhibited the capability of *S. mutans* for biofilm formation and enamel demineralization based on carious lesion model *in vitro* [10, 11]. In addition, some sweeteners have shown a decrease in bacterial adherence and biofilm mass production compared with sucrose based on a mixed inoculation of *S. mutans*, *S. sanguinis*, and *S. mitis* on glass beads [9]. However, it is still unknown how sugar substitutes influence the cariogenic potential of oral biofilms closer to the real situation, as well as the competitive advantage between acidogenic pathogens and commensal bacteria. The present study has for the first time, uncovered the effect of NNSs on biofilm formation and its EPS/acid production from an ecological perspective in dual-species and saliva-derived multispecies models.

Initially, we observed that on pure cultivation, NNSs not merely prohibited the growth and acid production of *S. mutans*, but also that of *S. sanguinis*, indicating the necessity to investigate the influence of NNSs on interactions between different species and the collective cariogenic potential of oral biofilms. Since the phenomenon of antagonism between *S. mutans* and *S. sanguinis* is a typical *in vitro* microcosm of interactions between acidogenic and alkali-generating bacteria [24, 25], we employed the commonly utilized dual-species model comprised of *S. mutans* and *S. sanguinis* to study bacteria interplay. The results indicated that the metabolism of dual-species biofilm to produce acids was active in high sucrose condition, and the aciduric *S. mutans* made competitive predominance over *S. sanguinis* (with their biomass ratio close to 2). Additionally, the pH value of dual-species biofilms with NNSs was higher than that of sucrose, and the *S. mutans/S. sanguinis* ratio significantly declined to about 1, suggesting the inhibitory effects of NNSs on biofilm acid production and *S. mutans* advantage. These outcomes demonstrated the ecological benefit of NNSs on the perspective of the *in vitro* competition between acidogenic and alkali-generating bacteria.

To further identify the influence of NNSs on overall biofilm cariogenic potential, we established the saliva-derived multispecies biofilm model. Acid production, acidogenic/alkali-generating bacteria biomass ratio, and EPS generation are critical evaluation indicators for the cariogenic ability of biofilms, and each of them has a close association with others. *S. mutans* and other acidogenic organisms can metabolize fermentable sugars to produce acids which are responsible for cariogenesis [26, 27]. EPS generated by exoenzymes from *S. mutans* are considered as essential virulence factors of dental caries, as they form the core of the scaffold of cariogenic biofilms, provide abundant primary binding sites, and enhance bacteria cohesion [5]. EPS and other materials in the matrix could restrict the capability of saliva for neutralizing acids, contributing to the formation and maintenance of acidic microenvironments [28]. The acid microenvironment may induce decreased abundance of *S. sanguinis* which is more sensitive to acidic condition [29]. In contrast, as the most prevalent species contains the arginine deiminase system, *S. sanguinis* is capable of producing alkali to gain a competitive advantage over *S. mutans*, thus preserving pH homeostasis and protecting against caries [30, 31]. This study revealed that NNSs could significantly reduce the biofilm formation, EPS production, and *S. mutans*/*S. sanguinis* ratio, as well as increasing biofilm pH. Although the decline of *S. mutans*/*S. sanguinis* ratio primarily exhibited as an inclination (only acesulfame-K group significantly distinguished from sucrose group), the results of our experiments collectively provided elementary evidence for NNSs' effect of diminishing biofilm cariogenic capacity.

It is worth noting that there were some differences in the results of NNSs' effect on bacterial growth between the single-species biofilm model and the multispecies biofilm model. When cultured separately with NNSs, the biomass of *S. mutans* biofilms was higher than that of *S. sanguinis*, while coculture and culture with saliva-derived bacteria did not show obviously increased growth of *S. mutans relative* to *S. sanguinis.* We speculate one of the reasons is that the stronger capacity for EPS production of *S. mutans* made the single-species biofilms more adhesive against PBS rinses to remove the excess CV. Moreover, since the interaction between different bacteria strains may influence the ultimate effect of external substances, it is necessary to apply the biofilm model comprised of a more complex microbial community. However, it is still important to highlight that the results of the present study were attained by *in vitro* approach. Despite the fact that the multispecies model derives from human saliva and consists of much more species than selected bacteria [32], the batch culture with constant treatment was insufficient for mimicking the environment of the oral cavity and the daily consumption of NNSs. Moreover, it should be noted that sweeteners are not usually commercialized in their pure form, so the results cannot be directly extended to sugar substitutes in the market when interpreting the data. The stronger evidence for the ecological benefit of NNSs on oral microbiota needed to be provided by well-designed cohort studies with the subjects sustainably exposed to NNSs. Additionally, some research has reported that certain NNSs induce alterations of the gut microbiome and result in metabolic impairments of the host [33–35]. Therefore, whether NNSs can be metabolized by oral bacteria and the further influence of the interactions on the host are required to be comprehensively explored. As NNSs have diverse chemical structures and properties, it is conceivable that the metabolization of various NNSs has different ecological effects in biofilms. Since some natural extract has been identified as an alternative treatment in caries [36, 37], the effect of natural intense sweeteners such as steviol glycosides and thaumatin deserves further exploration.

## 5. Conclusion

Taken together, this study demonstrates that NNSs (acesulfame-K, aspartame, saccharin, and sucralose) suppress the growth and biofilm formation of *S. mutans* or *S. sanguinis* in planktonic culture and single-species biofilm. These sweeteners decrease *S. mutans*/*S*. *sanguinis* ratio and induce less acid/EPS production than sucrose in dual- and multispecies biofilm models. The benefit of NNSs for bacterial equilibrium in biofilm may have a promising prospect in caries management.

## Figures and Tables

**Table 1:** Concentrations of sucrose and NNSs in growth media.

Sweeteners	Relative sweetness	Molar mass (g/Mol)	Concentration (g/100 ml)	Concentration (Mol/L)
Sucrose	1	342.3	1	2.92E-02
Acesulfame-K	200	201.24	5.00E-03	2.48E-04
Aspartame	200	294.3	5.00E-03	1.70E-04
Saccharin	300	183.18	3.33E-03	1.82E-04
Sucralose	600	397.64	1.67E-03	4.19E-05

## Data Availability

All data of the study are available in the article and can be solicited from the corresponding authors.
